# Supplementary material for: Regulation of Survivin Isoform Expression by GLI Proteins in Ovarian Cancer
Source: Cells. 2019 Feb 6;8(2):128. doi: 10.3390/cells8020128 (PMC6406444; doi:10.3390/cells8020128)
Supplement: Supplementary file 1 [file cells-08-00128-s001.zip › Supplementary figure legends.docx]

Figure S1: Western blots showing the protein expression of GLI1-3 proteins in SKOV-3 knock-out lines. (A) Expression of GLI1 in GLI1KO line. (B) Expression of GLI2 in GLI2KO line. (C) Expression of GLI3 in GLI3KO line.

Figure S2: Western blots showing the overexpression of GLI1-3 proteins in SKOV-3 cell line. (A) Overexpression of GLI1 protein. (B) Overexpression of GLI2 protein. (C) Overexpression of GLI3R protein. (D) Overexpression of GLI3 protein.

Figure S3: Association between c.-1547C>T and survivin isoform expression. (A) Association between c.-1547C>T genotypes and expression of isoform S 2α. (B) Association between c.-1547C>T genotypes and expression of isoform S 2B. (C) Association between c.-1547C>T genotypes and expression of isoform S 3B. (D) Association between c.-1547C>T alleles and expression of isoform S 2α. (E) Association between c.-1547C>T alleles and expression of isoform S 3B.

Figure S4: Association between c.9194G>A and survivin isoform expression. (A) Association between c.9194G>A genotypes and expression of isoform S WT. (B) Association between c.9194G>A genotypes and expression of isoform S 2α. (C) Association between c.-1547C>T alleles and expression of isoform S WT. (D) Association between c.-1547C>T alleles and expression of isoform S 2α.

Figure S5: Association between c.9386T>C and c.10611C>A and survivin isoform expression. (A) Association between c.9386T>C genotypes and expression of isoform S 2α. (B) Association between c.9386T>C genotypes and expression of isoform S 3B. (C) Association between c.10611C>A genotypes and expression of isoform S 2α. (D) Association between c.10611C>A genotypes and expression of isoform S 3B.

Figure S6: Association between c.-625G>C, c.-235G>A and c.221+209T>C and survivin isoform expression. (A) Association between c.-625G>C genotypes and expression of isoform S 2α. (B) Association between c.-235G>A alleles and expression of isoform S 3B. (C) Association between c.221+209T>C alleles and expression of isoform S 3B.
