# Supplementary material for: Regulation of Survivin Isoform Expression by GLI Proteins in Ovarian Cancer
Source: Cells. 2019 Feb 6;8(2):128. doi: 10.3390/cells8020128 (PMC6406444; doi:10.3390/cells8020128)
Supplement: Supplementary file 1 [file cells-08-00128-s001.zip › Supplementary tables.docx]

Table S1: Oligos used for cloning of sgRNA into pX330-U6-Chimeric_BB-CBh-hSpcas9 expression vector. PAM sequence is marked in bold.

| OLIGO NAME | SEQUENCE 5'-3' | POSITION RELATIVE TO ATG |
| --- | --- | --- |
| GLI1 FW oligo | CACCGCCCTTTTTGGTGATTCATC**TGG** | +46 TO +23 |
| GLI1 RV oligo | CCAGATGAATCACCAAAAAGGGCCAAA |  |
| GLI2 FW oligo | CACCGGGCCTCAGCCCCGCTGATG**TGG** | +59 TO +82 |
| GLI2 RV oligo | CCACATCAGCGGGGCTGAGGCCCCAAA |  |
| GLI3 FW oligo | CACCTCGAGTGGAGCACTTCACTA**TGG** | +75 TO +52 |
| GLI3 RV oligo | CCATAGTGAAGTGCTCCACTCGACAAA |  |

Table S2**:** Primer sequences for splice variant expression. Primers not from Antonacopoulou et al [8] were designed with Primer3 online tool (<http://bioinfo.ut.ee/primer3/>) [36].

| SPLICE VARIANT | SEQUENCE 5'-3' | REFERENCE |
| --- | --- | --- |
| S WT | F - TGACGACCCCATAGAGGAAC | - |
|  | R - TCCTTTGCATTTTGTTCTTGG |  |
| S 2α | F - AACTGGCCCTTCTTGGAG | [8] |
|  | R - ACTTACATGGGGTCGTCATC |  |
| S 2B | F - GATGACGACCCCATTGG | [8] |
|  | R - TTATGTTCCTCTCTCGTGATCC |  |
| S 3B | F - GCCAAGAACAAAATTGAGAGAG | [8] |
|  | R - AACAGACCCTGGCAAACATC | - |
| S Δex3 | F - GACGACCCCATGCAAAG | [8] |
|  | R - GTGGCACCAGGGAATAAAC |  |
| *TBP* | F - CACGAACCACGGCACTGATT | [35] |
|  | R - TTTTCTTGCTGCCAGTCTGGAC |  |

Table S3**:** Sequences of genotyping primers. Primer names of the fragments located in the regulatory regions are according to the major polymorphism present in the fragment.

| FRAGMENT NAME | SEQUENCE 5'-3' | TM/°C | SIZE/bp |
| --- | --- | --- | --- |
| rs3764383 | F - GGAGGAGAGAAAGGGAGGAA | 66 | 209 |
|  | R - ACCTCAAGTGATCTGCCTGC |  |  |
| rs8073903 | F - CCCCTGACTCCAGAAGGTG | 60 | 175 |
|  | R - TCAGACAGGAGAGCTTTACAGG |  |  |
| rs17878467 | F - GATTACAGGCGTGAGCCACT | 62 | 159 |
|  | R - GTGTGCCGGGAGTTGTAGTC |  |  |
| *BIRC5* Exon 1 | F - GACTACAACTCCCGGCACAC | 58 | 251 |
|  | R - CCTCCAAGAAGGGCCAGT |  |  |
| *BIRC5* Exon 1a | F - AGCCCTTTCTCAAGGACCAC | 58 | 264 |
|  | R – CTCGATGGGGACAAAGCAG |  |  |
| *BIRC5* Exon 2.1 | F - CACTCACGAGCTGTGCTGTC | 62 | 250 |
|  | R - GAAGCAATGAGGGTGGAAAG |  |  |
| *BIRC5* Exon 2.2 | F - CGATGGGCTTTGTTTTGAAC | 60 | 242 |
|  | R - CAGGGTCTGCTGATGTATTCTG |  |  |
| *BIRC5* Exon 2B | F - CCCTTCTCTGCCCTTAATCC | 64 | 147 |
|  | R - TAGTGGAGACGGGGTTTCAC |  |  |
| *BIRC5* Exon 3 | F - AGAGGTGCCATATGGGAATG | 62 | 642 |
|  | R - CATTGAACAGGGTTTGAGCA |  |  |
| *BIRC5* Exon 3B | F - CCCTGGATTTGCTAATGTGA | 60 | 229 |
|  | R - AGCTCTGCTCTTAACCACTGC |  |  |
| *BIRC5* Exon 4 | F - CTGGGAAGCTCTGGTTTCAG | 62 | 310 |
|  | R - CTGGTGCCACTTTCAAGACA |  |  |
| rs1042489 | F - TGCATGACTTGTGTGTGATGA | 60 | 161 |
|  | R - CCGTTTCCCCAATGACTTAG |  |  |
| rs2661694 | F - TGTATCATCCGGGCTCCTT | 60 | 159 |
|  | R - ACAGAGGCTGGAGTGCATTT |  |  |

Table S4: Association of genotype and allele frequencies between OC samples and healthy controls.

| *rs17887126* | *c.-235G>A* | *n (controls)* | *%* |  | *n (OC)* | | *%* | *p* |
| --- | --- | --- | --- | --- | --- | --- | --- | --- |
|  | GG | 72 | 97.30 |  | 35 | 87.50 | | 0.051* |
|  | GA | 2 | 2.70 |  | 5 | 12.50 | |  |
|  | AA | 0 | 0.00 |  | 0 | 0.00 | |  |
|  | G | 146 | 98.65 |  | 75 | 93.75 | | 0.053* |
|  | A | 2 | 1.35 |  | 5 | 6.25 | |  |

* borderline significant
