# Supplementary figures and images for: Regulation of Survivin Isoform Expression by GLI Proteins in Ovarian Cancer
Source: Cells. 2019 Feb 6;8(2):128. doi: 10.3390/cells8020128 (PMC6406444; doi:10.3390/cells8020128)

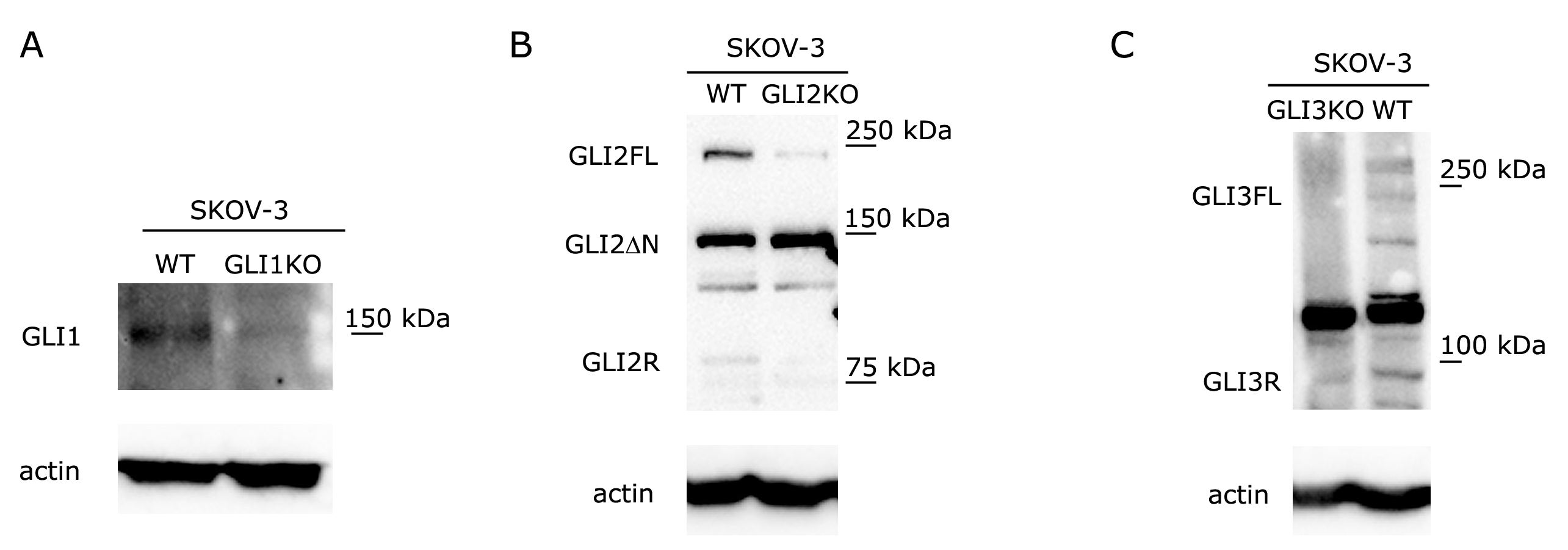

Supplement: Supplementary file 1 [file cells-08-00128-s001.zip › Supplementary figure 1.tif]

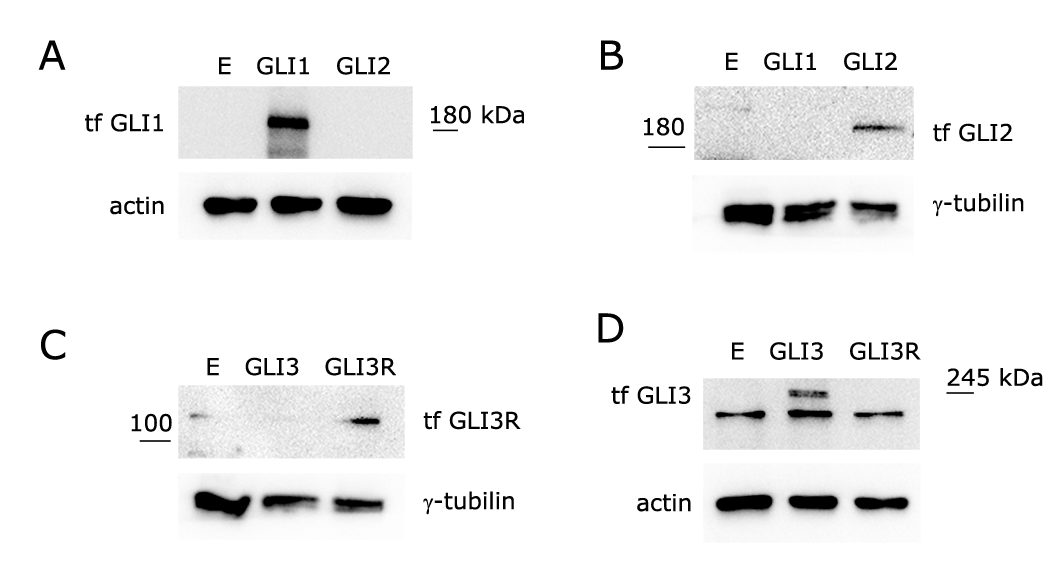

Supplement: Supplementary file 1 [file cells-08-00128-s001.zip › Supplementary figure 2.tif]

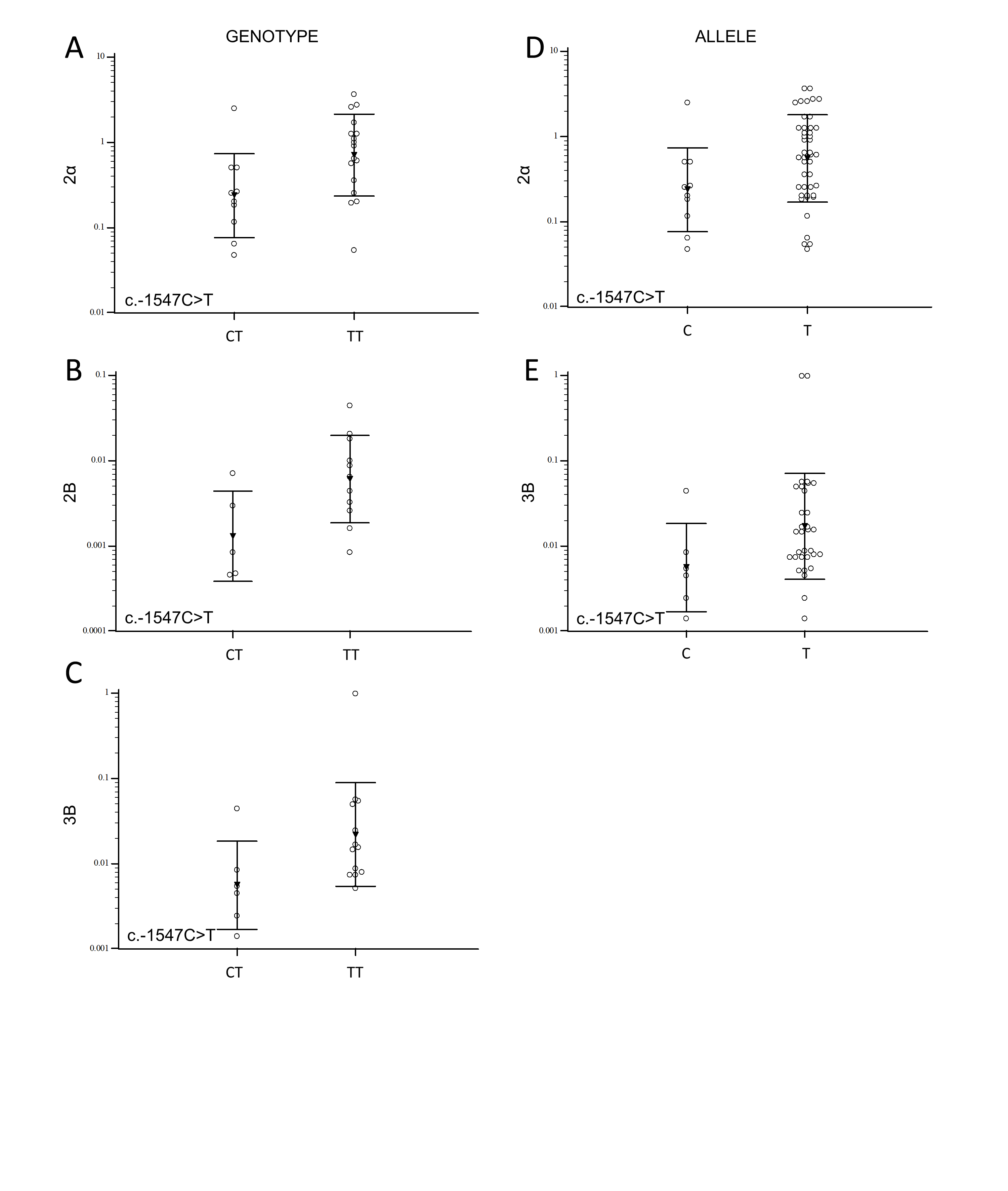

Supplement: Supplementary file 1 [file cells-08-00128-s001.zip › Supplementary figure 3.tif]

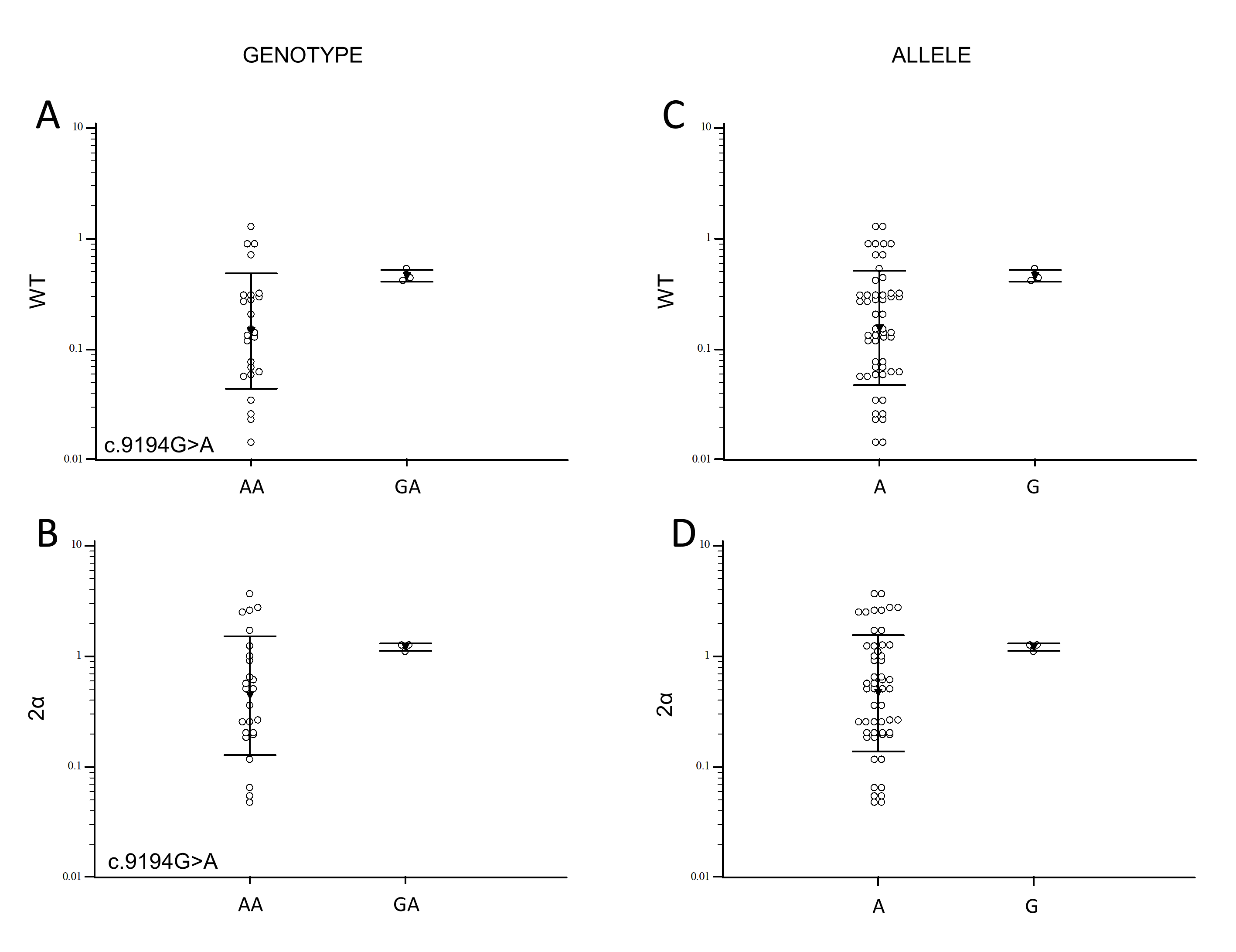

Supplement: Supplementary file 1 [file cells-08-00128-s001.zip › Supplementary figure 4.tif]

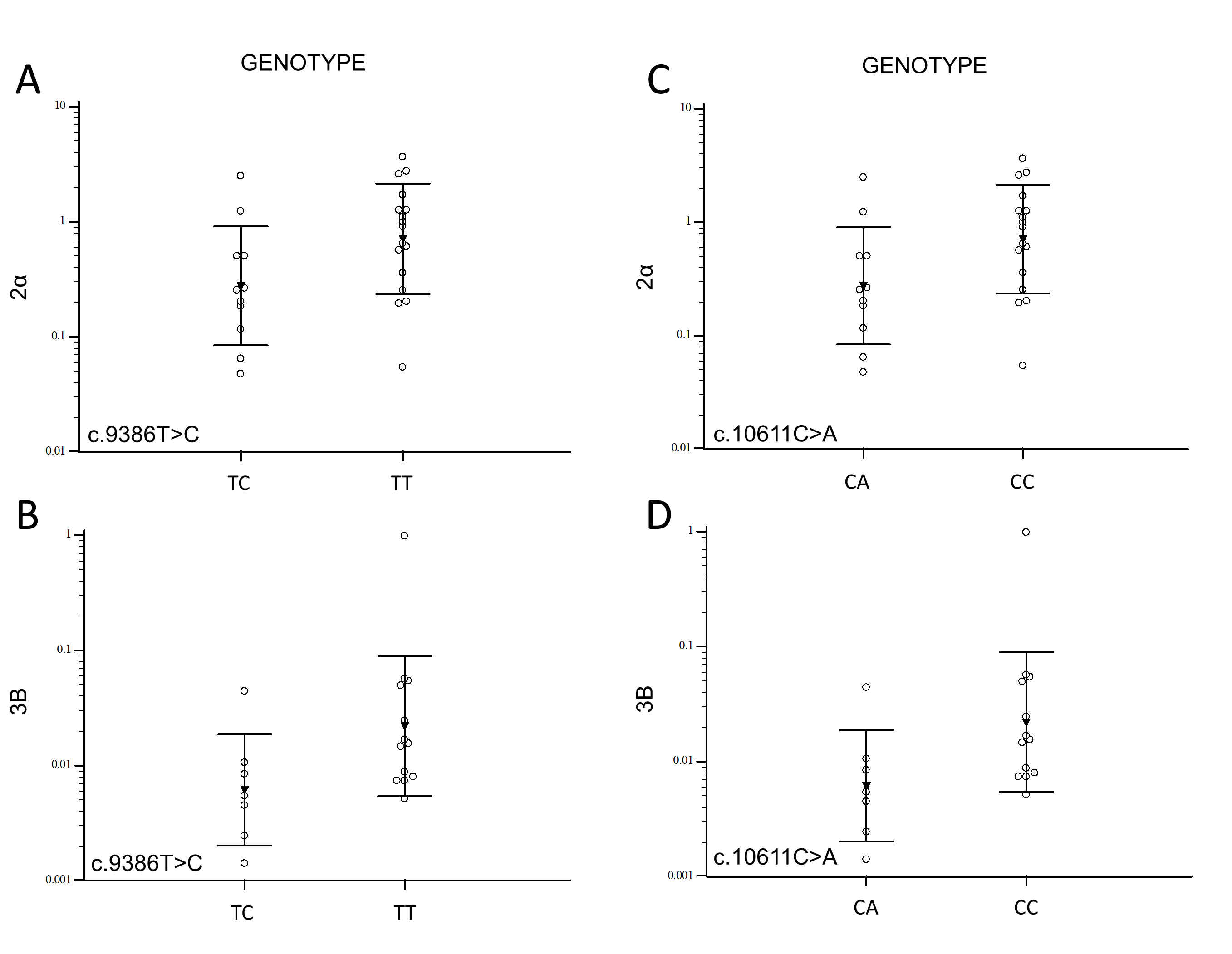

Supplement: Supplementary file 1 [file cells-08-00128-s001.zip › Supplementary figure 5.tif]

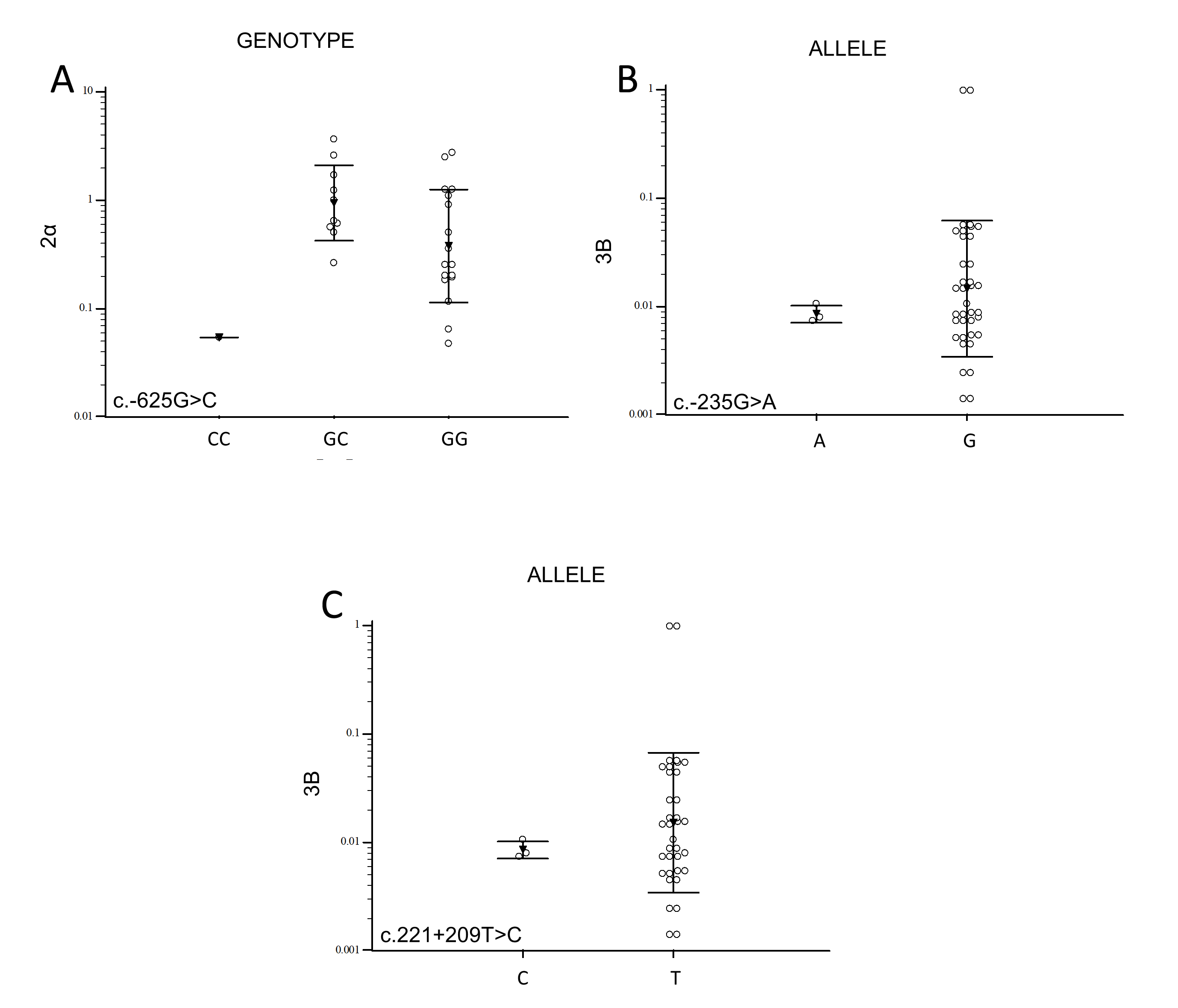

Supplement: Supplementary file 1 [file cells-08-00128-s001.zip › Supplementary figure 6.tif]
